# The Escalating Inequalities in Global Maternal Mortality—Time to Act: A Commentary

**DOI:** 10.1111/1471-0528.18246

**Published:** 2025-06-09

**Authors:** Anastasia Martin, Alexandra Ridout, Bosede B. Afolabi, Andrew Shennan

**Affiliations:** ^1^ Department of Women and Children's Health School of Life Course Sciences, Faculty of Life Sciences and Medicine London UK; ^2^ Department of Obstetrics and Gynaecology, College of Medicine University of Lagos/Lagos University Teaching Hospital Lagos Nigeria; ^3^ Centre for Clinical Trials, Research and Implementation Science University of Lagos Lagos Nigeria

**Keywords:** developing countries: obstetrics and gynaecology, epidemiology: general obstetric, maternal mortality, obstetric haemorrhage, pre‐eclampsia: clinical research

There are huge inequalities in maternal morbidity and mortality across the world, with low‐ and middle‐income countries (LMICs) reporting worse maternal outcomes. These disparities are not only stark, but are projected to get worse. LMICs account for 94% of all maternal deaths globally, with sub‐Saharan Africa (SSA) making up the majority (71%) of these cases [1]. The region's high birth rates, driven by limited access to contraception and inadequate family planning services, have resulted in a young and rapidly growing population of women of reproductive age [2]. If these trends continue unaddressed, maternal health inequalities will deepen over the next decade.

Global health inequalities are rooted in long‐standing structural inequities. The legacy of colonialism disrupted political and economic systems in many countries, contributing to ongoing challenges such as limited political freedom, unstable governments, and in some cases, corruption. Addressing these broader systemic issues should remain a global priority, requiring action at national and international levels. At the same time, we believe there is significant opportunity to improve women's health in the short and medium term through targeted interventions, even as longer‐term structural changes are pursued.

Sub‐Saharan Africa, home to 18% of the world's population, accounts for the vast majority of global maternal deaths, over 70% [3]. A woman in SSA is 24 times more likely to die from pregnancy‐related causes than a woman in Europe and five times more likely than her counterparts in Asia or Latin America [4]. This disproportionate burden stems from under‐resourced healthcare systems, limited access to skilled birth attendants, and cultural and socioeconomic challenges. This is further confounded by a young population (average age 19) and higher birth rates, so unless the disparities in care can be reversed, the inequalities in maternal mortality are likely to increase [5].

Figure 1 demonstrates the relative global proportions of population and maternal mortality in the world, along with the average age of the populations. The four regions highlighted represent 96% of the world's population. While global maternal deaths declined by 35% from 2000 to 2020, SSA saw only a 7% reduction during the same period—highlighting a growing disparity [4].

**FIGURE 1:**
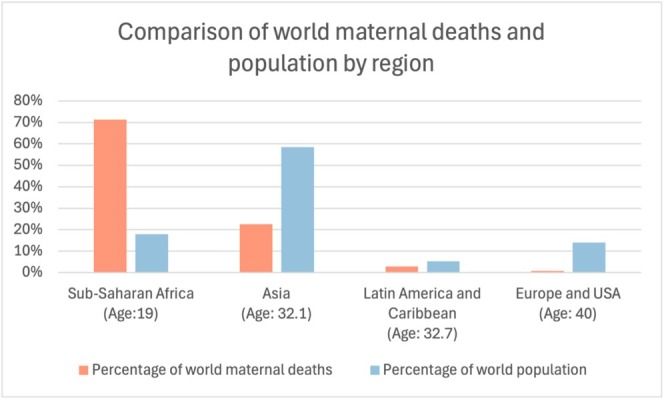
Comparison of world maternal deaths and population by relevant regions with Europe and United States of America (USA) as a comparator. Median age for each region noted in brackets.

The region's demographic profile compounds the challenge. With a median age of 19 compared to over 42 in Europe (United Nations Population Data), SSA faces increasing demands on maternal healthcare as its youthful population becomes reproductive. High birth rates, at 4.7 births per woman compared to 1.5 in Europe, correlate directly with surging maternal health demands [5, 6]. By 2100, nearly half of the world's children under five will reside in SSA [7]. Without significant investment in maternal health infrastructure, the strain on already fragile systems and maternal mortality rates will worsen, exacerbating disparities between high‐ and low‐income regions.

Addressing global maternal health inequalities requires immediate action and requires a collective response from multiple stakeholders. This includes both local and high‐income country governments, philanthropic organisations, academic institutions and global health bodies such as the World Health Organization (WHO). Funding to regions where the population of women of reproductive age is growing fastest should be prioritised. For example, high‐income countries can support this through overseas development assistance (ODA), while philanthropic organisations and global donors should also allocate targeted resources. These funds can support ministries of health to focus on strengthening healthcare infrastructure, training and retaining skilled birth attendants, and creating resilient healthcare systems including context‐specific evidence‐based interventions and capacity building tailored to local needs Improved access to contraception and family planning services can reduce high birth rates and empower women to make informed reproductive choices. Furthermore, funding to academic institutions and researchers, particularly in the countries most affected, should be increased to allow the generation of locally driven solutions.

The leading causes of maternal mortality—postpartum haemorrhage and preeclampsia—are preventable through simple, affordable interventions. Measuring blood pressure, detecting anaemia, and administering timely treatment are cost‐effective solutions that save lives. Yet, access to these basic diagnostics and interventions remains uneven in LMICs [8]. Addressing cultural barriers and fostering male partner involvement can further improve maternal health‐seeking behaviours and outcomes. It is pertinent to recognise that sub‐Saharan Africa represents a large spectrum of countries, cultures and beliefs. Effective implementation must be adapted to local contexts, considering diverse cultural practices, health systems and community needs. Community driven implementation is crucial to ensure long term and sustainable change in these diverse contexts.

The solutions are within reach. Clinical trials in low‐resource settings consistently demonstrate the effectiveness of simple interventions in reducing maternal deaths. For example, measuring blood pressure is an inexpensive screening tool that has been used to target action such as early delivery, and is still sometimes absent in healthcare facilities [8]. By instigating delivery, mortality can be significantly reduced while remaining cost‐effective [9]. What is missing is the widespread implementation and scale‐up of these life‐saving measures in the regions that need them most. Access to these cheap diagnostics and effective education of local healthcare staff can reverse these inevitable deaths without an increase in resource use and should be a major priority for funders. By focusing on the regions that account for the majority of maternal deaths and targeting the two leading causes, we can ensure we have the biggest impact.

Maternal health disparities are among the most extreme global inequities, yet they are also one of the most reversible. Immediate action to prioritise access to affordable, evidence‐based solutions in high‐burden regions can yield significant and rapid improvements. Ensuring a healthier future for mothers and their children should be a global priority, with the potential to address not only health outcomes but also broader social and economic inequalities. The time to act is now.

## Author Contributions

**A.M**.– original draft (lead), analysis of public data, writing. **B.B.A**.– review and editing. **A.R**.– original draft (supporting), review and editing. **A.S**.– conceptualisation, review and editing.

## Disclosure

The authors have nothing to report.

## Ethics Statement

This is a commentary based on previous studies and public data; therefore, no ethical approval is required.

## Conflicts of Interest

The authors declare no conflicts of interest.

## Data Availability

Data sharing not applicable to this article as no datasets were generated or analysed during the current study.
